# NGS‑based characterization of *Eimeria* spp. prevalence and diversity in broiler farms in northwestern São Paulo State, Brazil

**DOI:** 10.1590/S1984-29612026032

**Published:** 2026-07-31

**Authors:** Guilherme Zaratin Dumalakas, Bruna Matarucco Sampaio Beretta, Priscila Kataoka, Bruno Ferraz Itoyama, Brayan Kurahara, Marcelo Vasconcelos Meireles

**Affiliations:** 1 Universidade Estadual Paulista – UNESP, Faculdade de Medicina Veterinária, Araçatuba, SP, Brasil

**Keywords:** *Eimeria* spp., domestic chicken, molecular identification, Brazil, *Eimeria* spp., galinha doméstica, identificação, Brasil

## Abstract

The aim of this study was to determine the prevalence and diversity of *Eimeria* spp. in commercial broiler chicken farms (CBCFs) across 20 municipalities in northwestern São Paulo State, Brazil. Eighty-eight fecal samples corresponding to 88 commercial broiler farms were examined by microscopic screening for *Eimeria* spp. oocysts. All positive samples were subjected to genus-specific nested PCR (18S rRNA gene) followed by next-generation sequencing to identify the *Eimeria* spp. Microscopy detected *Eimeria* spp. oocysts in 100% (88/88) of the samples. Next-generation sequencing revealed the following order of prevalence of *Eimeria* spp. sequences: *Eimeria maxima*: 83/88 (94.3%; CI: (87.4–97.6); *Eimeria acervulina*: 75/88 (85.2%; CI: 76.4–91.2); *Eimeria necatrix/tenella*: 19/88 (21.6%; CI: 14.3–31.3); *Eimeria praecox*: 19/88 (21.6%; CI: 14.3–31.3); and *Eimeria mitis/mivati*: 10/88 (11.4%; CI: 6.3–19.7). No sequences corresponding to *Eimeria brunetti, Eimeria lata, Eimeria nagambie*, and *Eimeria zaria* were detected. In conclusion, the highest prevalences were observed for *E. maxima* and *E. acervulina,* whereas *E. tenella/necatrix, E. mitis/mivati*, and *E. praecox* showed lower prevalences. *Eimeria brunetti*, *E. lata*, *E. nagambie*, and *E. zaria* were not detected in CBCFs from northwestern São Paulo State.

## Resumo

Este estudo teve como objetivo determinar a prevalência e a diversidade de *Eimeria* spp. em núcleos comerciais de frangos de corte (NCFCs) da região noroeste do estado de São Paulo, Brasil. Oitenta e oito amostras fecais, correspondentes a 88 NCFCs, foram examinadas por microscopia para detecção de oocistos de *Eimeria* spp. Todas as amostras positivas foram submetidas à PCR nested específica para o gênero (gene 18S rRNA), seguida de sequenciamento de nova geração, para identificação de *Eimeria* spp. O exame microscópico detectou oocistos de *Eimeria* spp. em 100% (88/88) das amostras. O sequenciamento de nova geração revelou a seguinte ordem de prevalência de sequências de *Eimeria* spp.: *Eimeria maxima*: 83/88 (94,3%; IC: 87,4–97,6); *Eimeria acervulina*: 75/88 (85,2%; IC: 76.4–91,2); *Eimeria necatrix/tenella*: 19/88 (21,6%; IC: 14,3–31,3); *Eimeria praecox*: 19/88 (21,6%; IC: 14,3–31,3); e *Eimeria mitis/mivati*: 10/88 (11,4%; IC: 6,3–19,7). Não foram detectadas sequências correspondentes a *Eimeria brunetti, Eimeria lata, Eimeria nagambie* e *Eimeria zaria*. Em conclusão, as maiores prevalências foram observadas para *E. maxima* e *E. acervulina*, enquanto as menores foram observadas para *E. tenella/necatrix*, *E. mitis/mivati* e *E. praecox*. *Eimeria brunetti*, *E. lata*, *E. nagambie* e *E. zaria* não foram detectadas nas amostras examinadas.

## Introduction

Coccidiosis is a major disease in the poultry industry, with a high prevalence in domestic chicken farms. Infections involving multiple *Eimeria* species are frequently observed in industrial broiler operations (McDougald et al., 1987; Moraes et al., 2015; Beretta et al., 2024) as well as in alternative production systems (Godwin & Morgan, 2015; Terra et al., 2021; Soares et al., 2023).

Domestic chickens can be infected by 10 *Eimeria* species: *Eimeria acervulina*, *Eimeria brunetti*, *Eimeria lata*, *Eimeria maxima*, *Eimeria mitis*, *Eimeria nagambie*, *Eimeria necatrix*, *Eimeria praecox*, *Eimeria tenella*, and *Eimeria zaria* (Vrba et al., 2010, 2011; Blake et al., 2021).

Infections with *Eimeria* spp. can affect productivity parameters and increase mortality, depending on the specific species involved (Mesa-Pineda et al., 2021). Although limited information exists regarding infections with *E. lata*, *E. nagambie*, and *E. zaria*, current evidence suggests that commercially available vaccines offer no cross-protection against these species; additionally, these infections have been shown to negatively impact productivity and contribute to higher mortality rates (Morris et al., 2007; Fornace et al., 2013; Blake et al., 2021).

The primary preventive strategies against coccidiosis in broiler chickens involve the administration of anticoccidial drugs and vaccines (Mesa-Pineda et al., 2021). Due to the absence of interspecific cross-immunity, vaccines must incorporate all *Eimeria* species endemic to a given region, which can only be accurately determined through molecular biology methods (Blake et al., 2021).

Seven species of *Eimeria* from domestic chickens (*E. acervulina*, *E. brunetti*, *E. maxima*, *E. mitis*, *E. necatrix*, *E. praecox*, and *E. tenella*) have already been reported in Brazil in industrial broiler farms (McDougald et al., 1987; Carvalho et al., 2011; Moraes et al., 2015; Balestrin et al., 2021; Beretta et al., 2024). In contrast, all ten species infecting domestic chickens have been identified in alternative farming systems (Soares et al., 2023; Itoyama et al., 2025).

*Eimeria* species identification is usually performed through the analysis of oocyst morphology and morphometry, in addition to the evaluation of macroscopic lesions (Long & Joyner, 1984; Castañón et al., 2007). Nevertheless, these methods are subjective and may result in misdiagnosis, particularly because infections often involve multiple species (Frölich et al., 2013; Haug et al., 2008; Blake et al., 2021). Precise identification of *Eimeria* species in domestic chickens is most reliably achieved using species-specific PCRs (Vrba et al., 2010; Fornace et al., 2013; Blake et al., 2021; Jaramillo-Ortiz et al., 2023). However, next-generation sequencing has been employed for species-specific diagnosis and for the identification of novel operational taxonomic units (OTUs) of *Eimeria* (Hinsu et al., 2018; Hauck et al., 2019; Jenkins et al., 2025).

Most studies on the epidemiology of coccidiosis in domestic chickens have not investigated the presence of *E. lata*, *E. nagambie,* and *E. zaria*. To date, the only study conducted in Brazil employing next-generation sequencing to identify *Eimeria* spp. in broiler chickens from commercial farms did not detect *E. lata, E. nagambie*, or *E. zaria*. In contrast, *E. acervulina*, *E. maxima*, *E. mitis*, and *E. praecox*, species commonly reported in broiler production systems, were identified (Beretta et al., 2024).

The state of São Paulo accounts for 8.44% of Brazil’s broiler chicken production, corresponding to 0.46 billion of the 5.45 billion birds slaughtered nationwide in 2024 (ABPA, 2025). Despite the state’s importance to the national poultry sector, no studies have previously investigated industrial broiler farms in this region using diagnostic approaches capable of detecting all ten *Eimeria* species. Therefore, the aim of the present study was to determine the prevalence of *Eimeria* spp., including *E. lata, E. nagambie*, and *E. zaria*, in fecal samples collected from commercial broiler farms located in the northwestern São Paulo State.

## Material and Methods

This study was approved by the Ethics Committee on Animal Use (CEUA) of the São Paulo State University (UNESP), School of Veterinary Medicine in Araçatuba, SP, under Process CEUA 80/2021.

### Fecal samples

**Figure 1:**
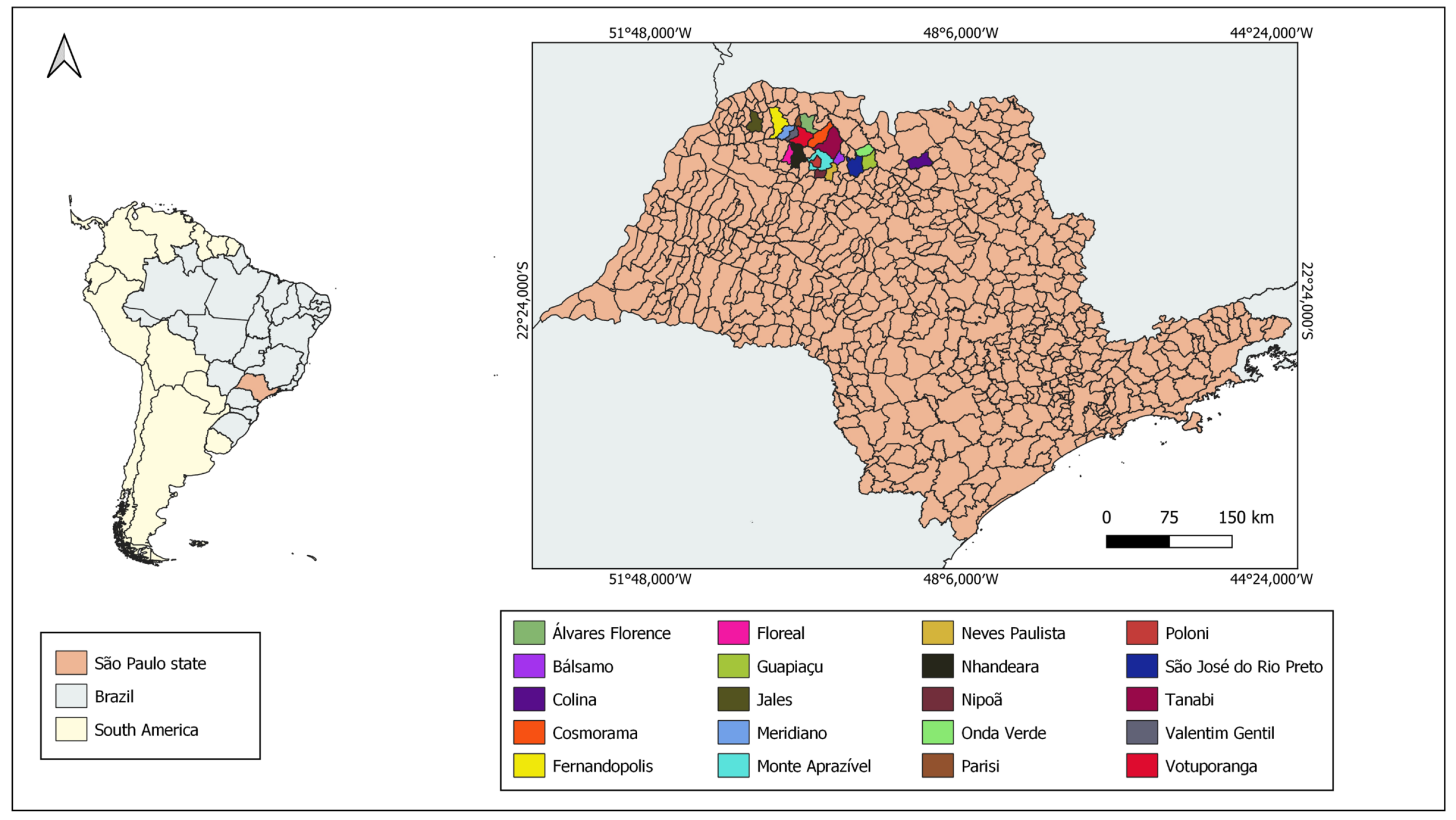
Locations of municipalities in northwestern São Paulo State, Brazil, from which chicken feces samples were collected in commercial broiler farms. The map was created using QGIS software version 3.28 (QGIS Development Team, 2023) and shapefiles from the Brazilian Institute of Geography and Statistics (IBGE, 2023).

The surveyed commercial broiler chicken flocks (CBCFs) are located in northwestern São Paulo State (Figure 1). Most CBCFs comprise one to three houses, with an average of about two per flock. These houses typically have concrete floors and wood-shavings litter and accommodate approximately 30,000 birds each. The houses within each CBCF are spaced about 10-15 meters apart, and the birds housed in these units are of the same age and origin, receiving standardized nutritional and sanitary management.

In most houses, litter is reused for three to eight production cycles following in-house composting, in accordance with company-specific protocols. New flocks are introduced after an average downtime of 15 days, during which the facilities are cleaned and disinfected. The most commonly administered anticoccidial agents include monensin, salinomycin, diclazuril, and maduramycin. None of the flocks were vaccinated against coccidiosis.

Sample sizes were calculated using the same methods described by Beretta et al. (2024), in accordance with the guidelines proposed by Sergeant (2018). The criteria for calculating the number of CBCFs, flocks per CBCF, and samples per flock included an expected prevalence of 50%, a margin of error of 10%, and a confidence level of 95%. The Coordination of Animal Health Defense, Secretariat of Agriculture and Supply of the State of São Paulo reported that 513 CBCFs were registered in the northwest region of São Paulo State at the time of sampling. Therefore, a minimum of 82 CBCFs was needed, and 88 samples, each representing a single CBCF, were collected in 20 municipalities during 2022 (Figure 1).

Samples were manually collected using a disposable wooden spatula by walking the entire house in a W-shaped pattern and subsequently kept in a 2.5% potassium dichromate solution at 4 °C for one to two months post-collection. Each sample comprised five pools, with each pool containing freshly excreted feces from 10 birds aged 21 to 35 days.

Fecal sample pools were processed individually following homogenization with deionized water containing 0.1% Tween 20 and subsequently passed through a metal sieve with a 600 µm pore size. The filtrates were then centrifuged at 2,000 × g for 10 minutes, and the resulting pellets were subjected to purification by centrifugal flotation in Sheather’s solution. The purified sediments from each of the five pools per flock were then combined to form a single composite sample. From this composite, two aliquots were prepared: one was preserved in 2.5% potassium dichromate at 4 °C for microscopic oocyst screening, and the other, comprising 150 mg of sediment, was stored at −20 °C for subsequent genomic DNA extraction.

### Microscopic screening for *Eimeria* spp. oocysts

All fecal samples were screened by microscopy for the detection of *Eimeria* spp. oocysts.

### Nested PCR and next-generation sequencing for *Eimeria* spp. identification

Genomic DNA was extracted from 150 mg of the sediment obtained after oocyst purification using the Quick-DNA™ Fecal/Soil Microbe Miniprep Kit (Zymo Research), following the manufacturer’s instructions. The resulting DNA samples were stored at -20 °C.

DNA samples were subjected to nested PCR using genus-specific primers to amplify a 260-bp fragment of the 18S rRNA gene (including the primer sequences) for subsequent next-generation sequencing (Hauck et al., 2019; Table 1). Both PCR rounds were performed using JumpStart™ Taq ReadyMix (Sigma-Aldrich) in a 25-μL final volume. First-round reactions contained 12.5 μL of ReadyMix, 400 nM of each primer, and 2.5 μL of template DNA. Nested-round reactions comprised 12.5 μL of ReadyMix, 800 nM of each primer, and 1.0 μL of the first-round amplicon as the template.

**Table 1:** PCR Primers for detection and identification of *Eimeria* spp. by nested PCR and next-generation sequencing in commercial broiler chicken farms.

**Primer**	**Primer sequence**	**Amplicon size (bp)**	***Eimeria* species**	**Target gene**	**Reference**
18S-F-out	CGGGTAACGGGGAATTAGGG	538	*Eimeria* spp.	18S rRNA	Hauck et al. (2019)
18S-R-out	TACGAATGCCCCCAACTGTC				
18S-F-in*	tcgtcggcagcgtcagatgtgtataagagacagATTGGAGGGCAAGTCTGGTG	260			
18S-R-in*	gtctcgtgggctcggagatgtgtataagagacagTGCTGCAGTATTCAGGGCRA				

* The Illumina adapter sequences are indicated in lowercase.

Samples were subjected to initial denaturation for 2 min at 94 °C followed by 35 cycles of denaturation at 94 °C for 30 s, annealing at 60 °C for 30 s, and extension at 72 °C for 30 s, followed by a final cycle at 72 °C for 7 min, using a SimpliAmp™ thermal cycler (Thermo Fisher Scientific). Genomic DNA extracted from oocysts from the commercial vaccine Bio-Coccivet R (Vaxxinova Biovet Brazil) and ultrapure water were used as positive and negative controls, respectively. Nested PCR amplicons were visualized by agarose gel electrophoresis, purified with a ProNex™ Size-Selective Purification System (Promega), and quantified using a Qubit™ digital fluorimeter (Thermo Fisher Scientific).

Next-generation sequencing was performed according to the Illumina 16S metagenomic protocol (Illumina, 2013), with 150 bp paired-end reads, using a MiSeq™ Reagent kit v2 (Illumina). Libraries were prepared using a volume of 50 μl containing 1 μl of the nested PCR amplicon, 5 μl of Nextera XT™ index primer 1 (N7xx), 5 μl of Nextera XT™ index primer 2 (S5xx), 25 μl of Kapa™ Hot Start High Fidelity Ready Mix (Kapa Biosystems), and 14 μl of ultrapure water. Samples were denatured at 95 °C for 3 min, followed by 8 cycles of denaturation at 95 °C for 30 s, annealing at 55 °C for 30 s, and extension at 72 °C for 30 s, with a final extension cycle at 72 °C for 5 min. Libraries were purified using the ProNex™ Size-Selective Purification System (Promega), quantified with a Qubit™ digital fluorimeter (Thermo Fisher Scientific), and subsequently normalized to a final DNA concentration of 6 pM. A PhiX control library was added at a proportion of 15%.

Library sequencing was performed at the Laboratory of Epigenomics of the College of Veterinary Medicine, UNESP, Araçatuba Campus, in a MiSeq™ platform (Illumina). Adapter sequences were trimmed following the Illumina FASTQ file generation pipelines included in the Illumina Experimental Manager software.

NGS data were processed using the Galaxy platform hosted on the public server *usegalaxy.org* (Afgan et al., 2018; The Galaxy Community, 2022). The analytical workflow included the following steps: 1) Fastp (Chen et al., 2018), employed for the preprocessing of FASTQ files and removal of low-quality reads (Q < 30); 2) Make.contigs (Schloss et al., 2009), used for primer trimming, paired-end read assembly, and generation of consensus sequences; 3) TN-93 clustering (Pond et al., 2005), used to merge matching reads into consensus clusters; and 4) Chimera.uchime (Schloss et al., 2009; Edgar et al., 2011), applied for the identification and removal of chimeric sequences.

Sequences were aligned to the corresponding reference sequences using the BioEdit™ Alignment Editor and subsequently compared with *Eimeria* spp. sequences through the Basic Local Alignment Search Tool (BLAST). Sequence validation followed three criteria: 1) sequences should form a distinct cluster; 2) the resulting consensus sequence had to exhibit at least 97% similarity to the reference sequence; and 3) the sequences representing that cluster should account for more than 1% of the total sequences within the sample.

### Statistical analysis

The 95% confidence intervals (CI) for prevalence estimates were calculated using the Wilson score method (Brown et al., 2001), as implemented in the Epitools Epidemiological Calculators (Sergeant, 2018).

## Results

Microscopy detected *Eimeria* spp. in 100% (88/88) of the samples. All samples were subsequently analyzed by nested PCR and next-generation sequencing, yielding the identification of the following prevalence rates of *Eimeria* spp. sequences: *E. maxima*: 83/88 (94.3%; CI: 87.4–97.6); *E. acervulina*: 75/88 (85.2%; CI: 76.4–91.2); *E. necatrix/tenella*: 19/88 (21.6%; CI: 14.3–31.3); *E. praecox*: 19/88 (21.6%; CI: 14.3–31.3); *E. mitis*/*mivati*: 10/88 (11.4%; CI: 6.3–19.7). No sequences corresponding to *E. brunetti*, *E. lata*, *E. nagambie*, and *E. zaria* were detected.

Although the *E. mivati* 18S rRNA gene is currently regarded as a type within the *E. mitis* genome (Vrba et al., 2011), in this study sequences were classified according to the species designations available in GenBank. *Eimeria necatrix* and *E. tenella* could not be differentiated by NGS of nested PCR amplicons.

All sequences generated in this study showed 100% genetic identity with *Eimeria* spp. reference sequences available in GenBank, including those previously reported in Brazil (Soares et al., 2023; Beretta et al., 2024) and in other countries (Schwarz et al., 2009). The matched reference sequences were as follows: *E. acervulina* (one sequence: OR226405, Brazil), *E. maxima* (two sequences: OR111213, Brazil; FJ236360, USA), *E. mitis* (one sequence: OR226409, Brazil), *E. mivati* (one sequence: OR226406, Brazil), *E. necatrix* (one sequence: DQ136185, China), *E. praecox* (one sequence: OR226407, Brazil), and *E. tenella* (three sequences: DQ136184 and DQ136177, China; KT184354, Canada).

Single infections with *E. acervulina* or *E. maxima* were detected in 12 of 88 (13.7%) samples. Mixed infections with *Eimeria* spp. were observed at the following frequencies: two species (39/88; 44.3%); three species (34/88; 38.6%); and four species (4/88; 4.5%) (Table 2).

**Table 2:** Identification of single or multiple infections with *Eimeria* spp. in commercial broiler chicken farms located in northwestern São Paulo State, Brazil.

**Eimeria species**	**No. broiler farms/ No. sampled (% positive)** **(% positive)**	**Confidence interval**
*E. acervulina + E. maxima*	33/88 (37.5)	28.1-47.9
*E. acervulina + E. maxima + E. necatrix/E. tenella* *	16/88 (18.2)	11.5-27.5
*E. acervulina + E. maxima + E. praecox*	12/88 (13.6)	8-22.3
*E. maxima*	10/88 (11.4)	6.3-19.7
*E. acervulina + E. maxima + E. mitis* **	5/88 (5.7)	2.5-12.6
*E. acervulina* + *E. maxima* + *E. mitis* + *E. praecox*	4/88 (4.6)	1.8-11.1
*E. acervulina*	2/88 (2.3)	0.6-7.9
*E. acervulina* + *E. mitis*	1/88 (1.1)	0.2-6.2
*E. acervulina + E. necatrix/E. tenella*	1/88 (1.1)	0.2-6.2
*E. acervulina* + *E. praecox*	1/88 (1.1)	0.2-6.2
*E. maxima + E. necatrix/E. tenella*	1/88 (1.1)	0.2-6.2
*E. maxima + E. praecox*	1/88 (1.1)	0.2-6.2
*E. maxima + E. necatrix/E. tenella + E. praecox*	1/88 (1.1)	0.2-6.2
Total	88/88 (100)	

* Sequences identified as either *E. necatrix* or *E. tenella* were interpreted as a single infection due to insufficient resolution for species differentiation;

** Sequences identified as *E. mivati* were classified as belonging to *E. mitis*.

## Discussion

We report a 100% prevalence of *Eimeria* spp. in broiler chicken farms. Previous epidemiological studies on coccidiosis in Brazilian CBCFs have similarly documented high prevalence levels of *Eimeria* spp., with reported rates ranging from 80.2% to 100% (McDougald et al., 1987; Carvalho et al., 2011; Moraes et al., 2015; Beretta et al., 2024).

There is a paucity of studies on poultry production systems in São Paulo State. Terra et al. (2001), using microscopy‑based diagnostics, reported *Eimeria maxima, E. brunetti, E. tenella, E. acervulina, E. mitis*, and *E. necatrix* in broiler chickens from Monte Alegre do Sul. More recent investigations across multiple municipalities in São Paulo State have identified ten *Eimeria* species in alternative domestic chicken systems using next‑generation sequencing (Soares et al., 2023; Itoyama et al., 2025). In this study, next‑generation sequencing detected five *Eimeria* species infecting broiler chickens; the highest prevalences were observed for *E. maxima* and *E. acervulina*, followed by *E. tenella/necatrix*, *E. praecox*, and *E. mitis*. No sequences corresponding to *E. lata*, *E. nagambie*, or *E. zaria* were identified. Higher prevalence rates of *E. acervulina* and *E. maxima* have been reported in most studies on coccidiosis in Brazilian broiler flocks (Moraes et al., 2015; Balestrin et al., 2021; Beretta et al., 2024) and in studies from other countries (Györke et al., 2013; Godwin & Morgan, 2015; Djemai et al., 2022; Flores et al., 2022; Cevallos-Gordon et al., 2024). In contrast, *E. tenella* has been reported as the predominant species in broiler flocks in several regions, including Pakistan (Awais et al., 2012), Nigeria (Jatau et al., 2016), India (Hinsu et al., 2018), and South Africa (Fatoba et al., 2020).

Although the NGS method used in this study cannot differentiate *E. necatrix* from *E. tenella*, the sequences detected most likely represent *E. tenella*, which is commonly reported in broiler chickens (Awais et al., 2012; Györke et al., 2013; Moraes et al., 2015; Balestrin et al., 2021; Andreopoulou et al., 2022; Liao et al., 2024). In contrast, due to a longer pre-patent period (Long et al., 1976) and lower fecundity compared to other *Eimeria* species (Bumstead & Millard, 1992), *E. necatrix* is usually associated with higher prevalence rates in long-lived chickens, such as commercial layers, backyard chickens, and breeders (Liao et al., 2024).

This study did not identify *E. lata*, *E. nagambie*, and *E. zaria* in CBCFs; however, their previous identification in various alternative farming systems in São Paulo State and in commercial chicken farms across multiple countries (Morris et al., 2007; Fornace et al., 2013; Godwin & Morgan, 2015; Clark et al., 2016; Jatau et al., 2016; Hinsu et al., 2018; Jaramillo-Ortiz et al., 2023) highlights the need for future research employing techniques capable of identifying all *Eimeria* species, particularly in breeder farms and free-range and cage-free commercial farms. Furthermore, rigorous sanitary protocols must be implemented to prevent the introduction of new *Eimeria* species in Brazilian commercial chicken farms.

## Conclusion

Using next-generation sequencing, five of the ten *Eimeria* species that infect domestic chickens were detected in CBCFs in northwestern São Paulo State. The species identified, in decreasing order of prevalence, were *E. maxima*, *E. acervulina*, *E. tenella/necatrix*, *E. praecox*, and *E. mitis/mivati*. In contrast, *E. brunetti*, *E. lata*, *E. nagambie*, and *E. zaria* were not detected in the broiler farms evaluated. Concurrent infections by multiple species were identified in most of the evaluated farms. Our findings underscore the potential of NGS as a highly sensitive and reliable tool for species-specific detection of *Eimeria* spp. and for the detection of infection with multiple species, offering critical insights for the development of more effective and targeted disease control strategies.

## Data Availability

The data supporting the findings of this study will be available upon request.
